# A case of meningitis due to varicella zoster virus reactivation in an immunocompetent child

**DOI:** 10.1186/1824-7288-39-72

**Published:** 2013-11-13

**Authors:** Susanna Esposito, Samantha Bosis, Raffaella Pinzani, Laura Morlacchi, Laura Senatore, Nicola Principi

**Affiliations:** 1 Pediatric Highly Intensive Care Unit, Department of Pathophysiology and Transplantation, Università degli Studi di Milano, Fondazione IRCCS Ca’ Granda Ospedale Maggiore Policlinico, Milan, Italy

**Keywords:** Herpes zoster, Varicella, Varicella zoster virus, VZV reactivation

## Abstract

**Background:**

The development of neurological complications due to varicella zoster virus (VZV) reactivation is relatively uncommon, particularly in the case of immunocompetent patients. Only a few cases have been described in the literature, most of which involved adult or elderly patients.

**Clinical presentation:**

Two days after his pediatrician had diagnosed herpes zoster and prescribed oral acyclovir 400 mg three times a day, a 14-year-old boy was admitted to our hospital because of mild fever, severe headache, slowness, drowsiness and vomiting. A cerebrospinal fluid examination was performed and showed an increased protein concentration (95 mg/dL), normal glucose level (48 mg/dL; blood glucose level, 76 mg/dL) and lymphocytic pleocytosis (1,400 lymphocytes/μL), and VZV DNA was detected by means of polymerase chain reaction (1,250 copies/mL). The results of immunological screening for HIV, lymphocyte subpopulation counts, serum immunoglobulin and complement (C3 and C4) levels, vaccine responsiveness and lymphocytes stimulation tests were unremarkable. Acyclovir was administered intravenously at a dose of 10 mg/kg three times a day and continued for 10 days. The therapy was highly effective and the patient’s clinical condition rapidly improved: fever disappeared after two days, and all of the signs and symptoms of neurological involvement after four days. The skin lesions resolved in about one week, and no pain or dysesthesia was ever reported. Given the favourable evolution of the illness, the child was discharged without further therapy after the 10-day treatment. The findings of a magnetic resonance examination immediately after the discontinuation of the antiviral therapy were normal, and a control examination carried out about four weeks later did not find any sign or symptom of disease.

**Conclusion:**

VZV reactivation can also lead to various neurological complications in immunocompetent children. Prompt therapy with acyclovir and the integrity of the immune system are important in conditioning outcome, but other currently unknown factors probably also play a role.

## Background

Varicella zoster virus (VZV) is an exclusively human neurotrophic alphaherpesvirus [1]. Primary infection causes varicella (chickenpox), after which the virus becomes latent in ganglionic neurons along the entire neuraxis. With advancing age or immunosuppression, cell-mediated immunity to VZV declines and virus reactivation causes herpes zoster (shingles) which can be complicated by central and peripheral nervous system involvement [2].

The development of the neurological complications of VZV reactivation is relatively uncommon, particularly in the case of immunocompetent patients. Only a few cases have been described in the literature, most of which involved adult or elderly patients [3-8]. Although there is no evidence concerning its efficacy, various antiviral regimens have been prescribed in such cases [2,9], including the administration of antiviral drugs not indicated for the pediatric population.

We here describe the case of an immunocompetent adolescent with herpes zoster and aseptic meningitis due to VZV reactivation because some of the patient’s clinical characteristics and the outcome of antiviral treatment make the case interesting.

## Case presentation

A 14-year-old boy was admitted to our hospital because of mild fever (axillary temperature 38°C), severe headache, slowness, drowsiness and vomiting. Two days before admission, his parents had noticed a maculopapular rash rapidly evolving into vescicles with an erythematous basis in a very limited region of the left dorsal skin. No other sign or symptom was reported. The patient’s pediatrician diagnosed herpes zoster and prescribed oral acyclovir 400 mg three times a day. The boy’s medical history was uneventful except for recurrent respiratory infections in his first three years of life. He was diagnosed as having varicella when he was three years old, but did not receive antiviral therapy.

Upon admission, his body temperature was 37.8°C, vital signs were unremarkable, and the skin lesions located in a small part of the area supplied with C8 were considered consistent with a diagnosis of herpes zoster. At the same time, a neurological examination revealed typical signs and symptoms of meningeal involvement: the patient was slightly confused and unable to tolerate bright light, his deep tendon reflexes were exaggerated, and he was positive for Brudzinski’s and Kernig’s signs. However, there was no sensory deficit. The results of routine blood examinations were within normal ranges, and there was no increase in inflammatory biomarkers. The findings of brain computed tomography (CT) and electroencephalography examinations were normal. A cerebrospinal fluid (CSF) examination showed an increased protein concentration (95 mg/dL), normal glucose level (48 mg/dL; blood glucose level, 76 mg/dL) and lymphocytic pleocytosis (1,400 lymphocytes/μL). VZV DNA was detected by means of polymerase chain reaction (PCR, 1,250 copies/mL), whereas the PCR analyses of herpes simplex virus 1 and 2, enterovirus, cytomegalovirus, Epstein Barr virus and JC virus were negative. The results of immunological screening for HIV, lymphocyte subpopulation counts, serum immunoglobulin and complement (C3 and C4) levels, vaccine responsiveness and lymphocytes stimulation tests were unremarkable.

The acyclovir dose was changed to 10 mg/kg three times a day, which were administered intravenously for 10 days. The therapy was highly effective and the patient’s clinical condition rapidly improved: fever disappeared after two days, and all of the signs and symptoms of neurological involvement after four days. The skin lesions resolved in about one week, and no pain or dysesthesia was ever reported. Given the favourable evolution of the illness, the child was discharged without further therapy after the 10-day treatment. The findings of a magnetic resonance examination immediately after the discontinuation of the antiviral therapy were normal, and a control examination carried out about four weeks later did not find any sign or symptom of disease Table 1.

**Table 1:** Characteristics of an immunocompetent adolescent with herpes zoster and aseptic meningitis due to VZV reactivation

**Characteristics**	**Data**
*Demographics*	
Age	14 years
Gender	Male
*Previous history*	
Age at time of developing varicella	3 years
Clinical problems	Recurrent respiratory tract infections in the first three years of life
*Clinical presentation upon admission*	
Axillary temperature	37.8°C
Skin lesions	Dorsal herpes zoster (C8)
Neurological symptoms and signs	Headache, slowness, drowsiness, unable to tolerate bright light, vomiting, stiff neck, exaggerated deep tendon reflexes, positive Brudzinski’s and Kernig’s signs
*Diagnostic examinations upon admission*	
White blood count	7,280/μL
Lymphocytes	31.7%
C-reactive protein	0.10 mg/dL
CSF examination	Protein 95 mg/dL, glucose 48 mg/dL, 1,400 lymphocytes/μL, PCR positive for VZV DNA 1,250 cp/mL, PCR negative for herpes simplex virus 1 and 2, enterovirus, cytomegalovirus, Epstein Barr virus, JC virus
Immunological screening	HIV negative, normal lymphocyte subpopulation counts, normal serum immunoglobulin and complement levels, vaccine responsiveness and lymphocytes stimulation tests
Electroencephalography	Normal
CT and MR	Normal
*Antiviral therapy*	
Oral acyclovir	400 mg 3 times a day for 48 hours (administered at home before neurological involvement)
Intravenous acyclovir	10 mg/kg 3 times a day for 10 days (administered after admission because of meningitis)
*Outcome*	
Duration of fever	2 days
Duration of neurological involvement	4 days
Duration of vescicular eruption	7 days
Duration of hospitalisation	10 days
Clinical evaluation after one month	Normal with absence of neurological involvement

CSF: cerebrospinal fluid; CT: computed tomography; MR: magnetic resonance; PCR: polymerase chain reaction; VZV: varicella zoster virus.

## Conclusions

Herpes zoster mainly affects elderly (its incidence is 8–10 times higher in people aged >60 years than in younger subjects) and immunocompromised patients, including those with acquired immunodeficiency due to drug administration [10], although bone marrow transplant recipients and HIV-seropositive patients are also at particular risk [11]. Waning cell-mediated immunity is thought to be a major factor in the increased incidence of herpes zoster because antibody titres remain unchanged or may even increase with age [2,11], which explains why there are only a few reported cases in immunocompetent children [1,3].

After varicella, VZV remains latent in the sensory dorsal root ganglia and, when reactivated, replicates along the course of the nerve and appears as a vesicular skin rash [1,2,9]. In most cases, this is preceded by paresthesia, itching and pain or “pre-herpetic neuralgia” [2,9], and these sensory symptoms can be severe enough to suggest an alternative cause, such as coronary ischemia or an abdominal condition. Pain and itching are the usual concomitants of the eruption, but they can also follow the rash and become chronic but self-limiting “post-herpetic neuralgia” [2,9]. Some atypical cases may lead to pain without any skin lesions, although a significant rise in VZV antibody titres suggests that the pain is closely related to VZV [2,9,10].

The situation in our patient was the opposite: although limited in extent, the skin lesions were clear and easily diagnosed as VZV-related alterations but, surprisingly, they were not associated with any significant pain or dysesthesia throughout the duration of the acute disease or later, including post-herpetic neuralgia. The child had no sensory neurological deficits that could explain the lack of pain in a disease that selectively damages sensory nerves. Consequently, it is reasonable to think that the benign clinical picture was related to the efficiency of his immune system despite the fact that VZV involved the central nervous system while he was receiving antiviral therapy. The positive evolution of the case could also have been due to the prompt acyclovir treatment, whivh is known to be very effective against VZV.

Aseptic meningitis is one of the possible complications of VZV reactivation. It is not one of the most severe neurological complications and usually does not lead to the major brain damage characterising VZV myelopathy, vasculopathy or eye disease [2]. In our case, although the neurological involvement was clinically evident, it was very mild and did not cause any persistent damage, possibly because oral antiviral therapy was started as soon as herpes zoster was diagnosed and, even if it could not have led to high acyclovir concentrations in the CSF, it may have attenuated the severity of the meningitis and favoured a positive clinical outcome. In this regard, this case resembles those recently described by Mantero *et al*. [4] and Goyal *et al*. [5], who respectively described VZV meningoencephaloradiculoneuropathy in an immunocompetent young woman with meningitis and cranial palsy and meningitis in a 27-year-old man, both of whom completely recovered. However, these patients received prolonged acyclovir therapy, whereas our patient required only 10 days of intravenous treatment. Further information should be collected concerning when and how long antiviral treatment should be administered to patients with neurological involvement due to VZV reactivation.

The findings of the immunological examinations of our child were normal, but a normal immune system does not guarantee the benign course of the neurological manifestations of VZV reactivation. Mpaka *et al*. have described the case of a previously healthy 66-year-old man whose obtundation deteriorated to coma within 24 hours of disease onset [6] after the development of lymphocytic meningitis with multiple, bilateral edematous and hemorrhagic lesions predominantly in the white matter, and intraventricular and subarachnoid hemorrhaging. The diagnosis of VZV encephalitis was confirmed by PCR analysis of the CSF, and he received adequate treatment with acyclovir. However, although his general condition was considered good two years later, this case shows that meningitis induced by VZV reactivation can have a complicated evolution even in immunocompetent subjects.

Our case underlines the fact that VZV reactivation can also lead to various neurological complications in immunocompetent children, which may be benign (as in our case) or very severe [2,10]. Prompt therapy with acyclovir and the integrity of the immune system are important in conditioning outcome, but other currently unknown factors probably also play a role. Further studies are needed to clarify the relationships between VZV and neural damage.

## Consent

Written informed consent to the publication of this case report and any accompanying images was obtained from the patient’s parents. A copy of the written consent is available for review by the Editor of the journal.

## Abbreviations

CSF: Cerebrospinal fluid; CT: Computed tomography; MR: Magnetic resonance; PCR: Polymerase chain reaction; VZV: Varicella zoster virus.

## Competing interests

The authors declare that they have no competing interests.

## Authors’ contributions

SE drafted the manuscript, supervised the management of the clinic, and was head of the pediatric clinic in which the patient was hospitalised; SB, RP, LM and LS admitted the patient, followed him during hospitalisation, and carried out the follow-up examinations; NP co-wrote the draft manuscript. All of the authors read and approved the final version of the manuscript.
